# Do medical interns publish findings of compulsory audit or research projects? Five-year experience from a single centre in New Zealand

**DOI:** 10.30476/jamp.2019.81894.1040

**Published:** 2020-04

**Authors:** YASSAR ALAMRI, KHALID ALSAHLI, JENNY BUTLER, TOM CAWOOD

**Affiliations:** 1 New Zealand Brain Research Institute, Christchurch, New Zealand; 2 Department of General Medicine, Christchurch Public Hospital, Christchurch, New Zealand; 3 The Prince of Wales Clinical School, University of New South Wales, NSW, Australia; 4 Department of Endocrinology, Christchurch Public Hospital, Christchurch, New Zealand

**Keywords:** Medical interns, Research projects, Compulsory audit

## Abstract

**Introduction:**

There is a paucity of literature on research output of Australasian interns. We have previously shown great interest among interns rotating in our department to publish or present their findings from an audit or research project (ARP). The aim of this study was to examine the output of the intern ARP.

**Methods:**

ARP titles over a five-year period were searched in academic databases. We compared the output rate from our institution to a rate estimated *a priori* from previously published literature.

**Results:**

A total of 186 ARPs were conducted over the study period. Of these, only two were published (one original article and one letter) and one was presented at a national conference. The observed productivity rate was significantly lower than that of the estimated rate (χ2 = 4.49, *p* = 0.034).

**Conclusion:**

Despite potential limitations, our study remains the largest study to report on intern research productivity in Australasia. It provides evidence of the need for improvement in and encouragement of research conducted by junior doctors.

## Introduction

### Intern research

Intern research refers to research that is conceptualised and carried out during internship year(s) by newly qualified doctors. Not only is conducting research pivotal in enhancing interns’ knowledge of the literature and critical analysis skills, it is becoming increasingly essential for career progression (and sometimes a requirement by licencing medical bodies ( 1 ). Subjecting research findings to peer-review and subsequently disseminating the results (through publication or presentation) serve as evidence of the rigour and quality of the research ( 2 ).

Given their workload, interns often place less priority to engaging research electively, and for compulsory research components, to disseminating the findings (i.e. publication or presentation) ( 3 ). This, unfortunately, deters intern participation in research, which, in turn, leads to reduced likelihood of future research involvement ( 4 ) and increased difficulty obtaining a position in specialist training programmes ( 3 ).

There is a paucity of literature on intern research output. Data from research done by interns and junior residents in training programmes with no dedicated research components demonstrate sub-optimal productivity with output ranging from 0.01-0.1 publications or presentations per junior resident per year ( 5 ). Extrapolating these results to intern research output, figures are likely to be lower because unlike residents, interns do not usually have protected research time despite a comparable workload.

### Interns in Australia and New Zealand

**Figure 1:**
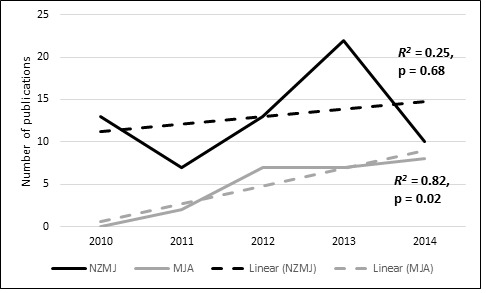
Figure 1. An increasing number of regional publications authored by Australasian interns in the MJA but not the NZMJ; MJA = Medical Journal of Australia, NZMJ = New Zealand Medical Journal.

During their first year, interns in Australia and New Zealand are required to complete predefined periods (e.g. a minimum of 10 weeks) in medical and surgical rotations at accredited hospitals ( 6 ). Whilst achieving certain clinical competencies is a prerequisite to obtaining general registration (i.e. practice licensure), research-related activities are usually institution- (or department-) dependent. Examining regional journals reveals a mixed trend in the number of publications authored by Australasian interns (Figure 1).

At our institution, interns rotating in the General Medicine department are required to complete an audit or research project (ARP), and present their findings at the morning handover meeting at the end of the rotation. Not only does the ARP serve to familiarise interns with the research process/audit cycle, but it is also hoped to enhance the intern’s understanding of the topic they are researching, as well as help progress their careers (e.g., through resultant publications or conference presentations). Participation in such scholarly activities has not only been shown to enhance trainees’ understanding of the topic ( 7 ), but also (at least in the case of medical students) potentiate their future academic productivity ( 8 ).

Each year, around 35-45 interns complete their General Medicine rotation in blocks of 13 weeks. During their rotation, interns are expected to complete a clinical audit (i.e., a study comparing the department’s current practice with best available standards/guidelines) or research project (i.e., a study that aims to find answers for novel questions).

The choice of project type (i.e., audit vs. research), topic and extent of data acquisition is left to the intern’s discretion. In addition, interns are allowed to combine efforts in order to conduct a more substantial ARP. Towards the end of their rotation, interns are required to present their findings during the department’s morning handover meeting.

We previously investigated the interns’ attitudes towards the General Medicine ARP component of their rotation over a two-year period. Most interns chose their own topic (65%), spent 5-10 hours conducting the ARP (63%) and felt encouraged to engage in further similar projects in the future (65%). The majority of interns (59%) saw the ARP as an opportunity to change practice in the department, although only 32% felt their findings would actually be practice-changing. However, the most common motivator for the ARP (74%) was the pursuit of publishing the findings. Therefore, the aim of this study was to examine the output of the intern ARP.

## Methods

### Study setting

This retrospective study included all ARPs presented by general medical interns between November 2009 and November 2014. For the purposes of this study, all projects completed by interns (whether audit or research) were included.

### Search strategy

Key words of project titles were searched using PubMed and Google Scholar databases. Findings were correlated with author names and affiliations. For conference proceedings, the Conference Proceeding Citation Index was utilised.

### Statistical analysis

Based on previous experience ( 5 ), we estimated an average output of 0.05 publication/presentation per intern per year. This gave an estimated output of 9.3 publications or presentations for the period studied.

A two-tailed chi-squared test (χ2) was used to test the actual research output against the estimated output. Statistical significance was determined if type I error rate was < 5% (p-value < 0.05). The analyses were performed using SPSS Statistics® software package (version 22.0.0.0).

### Ethical consideration

Ethical approval was not required as this was an electronic review of freely available material with no human interaction or intervention (clinical or otherwise).

## Results

### Intern and project characteristics

A total of 186 general medical interns were included in the study, with a female-to-male ratio of 1.3:1. In total, 130 ARPs were conducted: 76 (58.5%) by a single intern, 52 (40%) by two interns and 2 (1.5%) by three interns.

Common themes emerged from reviewing the ARPs. These, in descending order of frequency, included: pharmacology (e.g. prescribing behaviours; 22%), medical note documentation (10%), disease-specific projects (e.g. diabetic retinopathy; 9%), admission and discharge logistics (9%), neurosciences (e.g. stroke rehabilitation pathways; 8.5%), medical trivia (e.g. coffee consumption among resident doctors; 8%), venous thromboembolism (8%), intern and medical student wellbeing (7.5%), medical technology (5%), quality and safety (5%), patient lifestyle choices (e.g. smoking; 4%) and work-up investigations (e.g. pattern of radiology use; 4%).

### Project outcomes

Although no objective data exist, at least 1-2 ARPs per rotation (i.e., 4-8 per year) were deemed by the department senior staff to be worthy of publication. Searching the online databases, however, only retrieved two publications in peer-reviewed journals: one original paper (Chieng et al., 2015) (with the intern as the third author) and one letter to the editor (Howey & Chin, 2013) (with the intern as the first author). There was also one presentation at a national conference where the intern presented their findings in a poster format (Sia, 2011). Two authors were male (letter and presentation) and one female (original paper).

Out of 186 interns over a five-year period, only 3 published or presented their findings, giving an output rate of 1.6% (i.e. 0.016 publication/presentation per intern). The observed rate of 1.6% was significantly lower than the estimated 5% (χ2 = 4.49, *p* = 0.034).

## Discussion

The current research output of general medical interns doing a compulsory project at our institution is 0.016 publications or presentations per intern. This is significantly lower than our hypothesised rate based on previous literature on intern research output ( 5 ) and represents a “loss” of this knowledge to the public domain as well as “waste” in terms of the limited learning that could have been shared more widely. To the authors’ knowledge, this is the first study to specifically address intern research output in Australasia, and similar data on the subject from outside the US are generally lacking.

Interns are generally under significant pressure as they learn to apply theory to practice, attend to sick patients and acquire time-management skills. At the same time, however, they are also expected to prepare for training college applications. With the increasing demand for training positions without a matching increase in the number of positions offered, interns have to ‘stand out,’ which includes being involved in research and disseminating results through publications and conference presentations ( 3 ). While the reported low rate of publications may stem from a general lack of experience with research/journal submissions (e.g., lack of familiarity since medical school), previous studies have identified several factors that impede junior doctors’ research productivity ( 9 ). These barriers can generally be divided into barriers to conducting research and barriers to result dissemination.

The most commonly cited barriers to conducting research are the lack of interest and lack of time ( 9 , 10 ). Several strategies have been implemented to counteract the weaning enthusiasm for research among medical practitioners. One such approach has been a ‘positive reinforcement’ plan whereby otolaryngology residents at the University of Missouri are awarded points for each step along the research path ( 5 ). These points can later be converted to a monetary value to be used on educational expenses. This has resulted in more than tripling of the publication rates ( 5 ). To address the interns’ lack of time to pursue research, it is paramount to recognise the importance of ‘protected research time’. The Internal Medicine programme at the University of California, Davis (USA) has recently introduced a four-week academic rotation for interns to focus on research, which has resulted in significant increases in conference presentations and publications ( 11 ). At our institution, interns appeared to be sufficiently motivated ( 10 ). However, protected research time per se does not exist and interns have to fit ARP-related work in their ‘downtime’ (e.g. pre-call days) of the rotation.

Barriers to result dissemination, on the other hand, mainly revolve around two areas: the availability of dissemination avenues and the lack of senior support ( 12 ). Senior help is often required for project planning, technical support with data collection, statistical help once data are collected and guidance to synthesise and write the findings.

Most journals encourage submission from junior researchers, and we have previously reviewed the plethora of medical journals that cater for medical students and junior doctors ( 13 ). In the present study, we did not differentiate between quality improvement/audits and research topics. This is because the line(s) that separate these can at times be artificial and/or indistinct ( 14 ). At our institution, interns are given the chance to select a topic of their choice or choose from a list of pre-selected audit as well as research projects. Furthermore, “publishability” of a project does not appear to depend on its type (i.e., audit vs. research). Therefore, neither difficulty with project conception nor dissemination is believed to have differed between audit and research topics in our study.

Critical to successful journal or conference dissemination of intern research is the support and guidance of senior mentors. This often translates to committed and enthusiastic faculty instead of leaving interns to find mentors ad hoc. At our institution, the ARP is coordinated by one Consultant, who only has four hours dedicated time per week for the ARP programme. The interns are then left to liaise with one of the General Medicine Consultants, if needed.

Because most of our interns’ ARP falls within definable themes, we suggest streamlining ARP of a particular theme to one or two Consultant mentors (preferably with special interest in the topic). Not only is this hoped to optimise productivity, but it also ensures continuity of the audit/research cycle as projects can be continued longitudinally. This way, small contributions by several interns can be collated into a cohesive single project that is worthy of publishing or presenting.

Certain limitations and biases are inherent to this study, including the retrospective design. There are also potential deficiencies in searching intern publications and presentations, especially in non-indexed journals and proceedings. However, data from multiple sources were sought in order to corroborate the reported findings. The designated benchmark output rate (0.05 publications/presentations per intern per year) was based on only two previous studies ( 15 - 17 ); this was done in an attempt to compare our publication rate with an ‘educated guess’ rate. Finally, this study remains a single centre’s experience and further studies are required to corroborate (or refute) our findings.

## Conclusion

Despite the low output rate of the interns, our findings are hardly surprising. Most interns presumably had limited research experience, which emphasises the importance of research education during medical school. This issue, in particular, needs to be recognised by medical educators. We are currently undertaking another prospective study of whether medical students who become engaged in research at an early stage, also later remain involved as interns and residents.

Other potential reasons for the low rate include the interns being pre-occupied by the multiple demands of a busy and challenging year, having limited senior support and the reported limited time spent (5 to 10 hours) on the ARP project—a very short time to produce a publishable manuscript.

Our study provides compelling evidence for the need for support and encouragement of research conducted by junior doctors. Streamlining similar ARPs under the careful guidance of one or two interested senior staff (at an institutional level) and encouraging junior doctors to pursue disseminating their findings (at an individual level) are two suggested solutions. Future research ought to focus on whether implementing the suggested solutions produces tangible differences in junior doctor productivity.
